# In search of optimal cardioplegia for minimally invasive valve surgery

**DOI:** 10.1177/02676591211012554

**Published:** 2021-06-03

**Authors:** Sion Russell, Salman Butt, Hunaid A Vohra

**Affiliations:** 1 Department of Cardiac Surgery/Cardiovascular Sciences, Bristol Heart Institute, University of Bristol, Bristol, UK; 2 Department of Perfusion Sciences, St George’s Hospital, London, UK

**Keywords:** cardioplegia, minimally invasive cardiac surgery, antero-lateral thoracotomy, mini-sternotomy, custodiol, whole blood cardioplegia, del Nido cardioplegia

## Abstract

Cardioplegic solutions are used in cardiac surgery to achieve controlled cardiac arrest during operations, making surgery safer. Cardioplegia can either be blood or crystalloid based, with perceived pros and cons of each type. Whilst it is known that cardioplegia causes cardiac arrest, there is debate over which cardioplegic solution provides the highest degree of myocardial protection during arrest. Myocardial damage is measured post-operatively by biomarkers such as serum TnT, TnI or CK-MB. It is known that the outcomes of minimally invasive valve surgery are comparable to full sternotomy valve operations. Despite there being a wide diversity in use of different cardioplegic solutions across the world, this comprehensive literature review found no superiority of one cardioplegic solution over the other for myocardial protection during minimally invasive valve procedures.

## Introduction

Cardioplegia was developed in 1955 consisting of high concentrations of potassium citrate leading to cardiac membrane depolarization and reversible cardiac arrest.^1^ Cardioplegia is infused into the heart via a catheter to stop the heart. By stopping the blood circulating into the heart, cardiopulmonary bypass (CPB) allows for cardioplegia to be used, hence protecting the heart from ischaemia whilst also allowing other tissue to be perfused.^2^ Broadly, there are two different compositions of cardioplegia: blood-based (BC) and crystalloid cardioplegia (CC). BC is used due to close resemblance with normal physiological oxygen delivery, whilst CC reduces oxygen consumption during procedures and therefore reduce ischaemic damage.^3^ Commonly used BC include St Thomas’ (4:1 blood:crystalloid), Harefield cardioplegia solution (high and low strength), Microplegia MP, del Nido’s cardioplegia (DNC) (1:4 blood:crystalloid) and University of Wisconsin solutions, whilst popular CC solutions include Bretschneider’s/Custodiol solution.^1,4^

Despite the use of laparoscopic tools to perform minimally invasive procedures being present as early as the 1950’s, minimally invasive cardiac procedures (without completely opening up the sternum to gain access to the thorax) have only been performed since the 1990’s.^5,6^ Now, minimally invasive cardiac surgery (MICS) techniques have advanced so that the surgery can be performed through a right or left mini-thoracotomy, or via a mini-sternotomy, whereby a small incision is made through the sternum, but does not go the entire length of the sternum.^5,7,8^ Whilst not gaining full access to the thoracic cavity by fully separating the sternum can present challenges to a surgical procedure, minimally invasive surgeries produce comparable results to a full sternotomy. These procedures may be preferred to a full sternotomy for enhance patient satisfaction and/or where poor prognostic factors may impair recovery.^5^ To undergo minimally invasive valve surgery (MIVS), CPB is needed and aortic occlusion is a critical step in its setup, which can be achieved by several techniques available to surgeons, including transthoracic clamp (TTC).^9^

Whilst cardioplegia is efficacious in causing cardiac arrest, there is discussion about which solution provides the highest level of myocardial protection, especially in MIVS. Highlighted by Ali et al, there is wide variation in the universal use of cardioplegia solutions which points towards lack of consensus regarding the safety of various solutions compared to others, calling for more research into the topic.^1,4^ This paper discusses the use of different cardioplegia solutions in MIVS, aiming to determine the superiority of one over the other.

## Method

**Figure 1.**
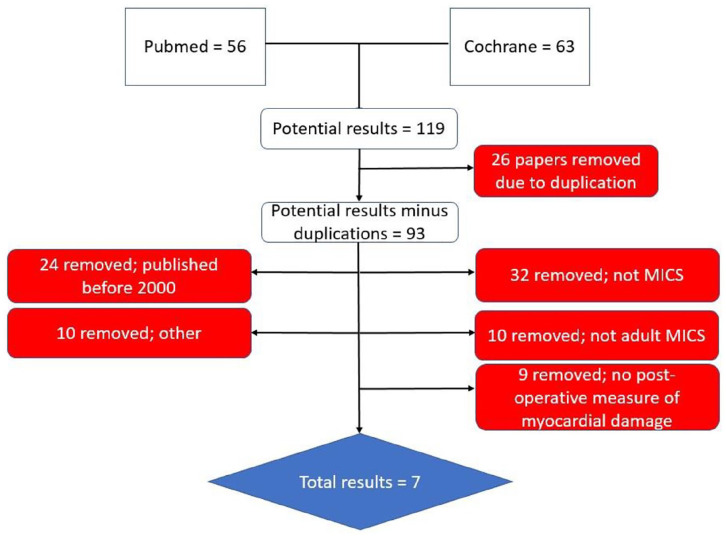
The flow chart showing the search results of the literature review and the criteria that resulted in exclusion of papers.

The medical search engines PubMed and Cochrane were used to identify sources for this review. Due to the solutions being the most widely used around the world, Bretschneider’s, DNC and St Thomas’ cardioplegic solutions were included in the search criteria, which is shown in Table 1. To be included in this work, there had to be discussion of which MIVS was performed, there had to be a comparison of at least two cardioplegic solutions, the cardioplegic solutions used must be named and the biomarkers used as a measure of myocardial damage should be defined (e.g, Troponin-T/-I (TnT/TnI respectively) or Creatine Kinase myocardial band (CK-MB)). Whilst both TnI and TnT are both known to be useful biomarkers for myocardial damage, TnI has not been identified outside of the cardiac myocytes, whereas TnT has been identified to a smaller extent in skeletal muscle. However, as they are both accurate markers for myocardial damage, they have both been included in search criteria.^10^ The results of the search criteria are shown in Figure 1. The literature search produced seven results,^7,11–16^ which are shown in Table 2. Two of the studies are single-centre that specify the same surgeon/surgical team performed the operations.^13,14^ The other five either specify the use of two or more surgical teams or do not specify how many surgical teams were used, which may lead to individual variation within the studies.^7,11,12,15,16^

**Table 1.** The search criteria used on PubMed and Cochrane databases.

Search number	Search term
1	Minimally invasive cardiac surgery [MeSH terms]
2	Bretschneider’s cardioplegia [MeSH terms]
3	Custodiol cardioplegia [MeSH terms]
4	Del Nido cardioplegia
5	St Thomas 2 cardioplegia
6	Blood cardioplegia [MeSH terms]
7	Troponin
8	CK-MB
9	1 + (2 OR 3) + 7
10	1+ (2 OR 3) + 8
11	1 + 4 + 7
12	1 + 4 + 8
13	1 + 4
14	1 + (5 OR 6) + 7
15	1 + (5 OR 6) + 8
16	1 + (5 OR 6)
17	1 + 6
18	(2 OR 3) + 4
19	(2 OR 3) + (5 OR 6)
20	4 + (5 OR 6)

Search numbers 9 to 20 are combinations of searches 1 to 8 to produce the search results.

**Table 2.** The papers included following the literature search.

Author	Cardioplegia(s) used	Minimally invasive surgery	Minimally invasive technique
De Palo M^12^	Bretschneider’s	AVR	Right anterolateral thoracotomy
	St Thomas’	MVR	
		Dual valve surgery	
Luo H^14^	Del Nido	AVR	Right anterolateral thoracotomy
	Whole blood	MVR	
		Dual valve surgery	
Matzelle SJ^13^	Custodiol	MVR	Right anterolateral thoracotomy
Mork C^7^	Bretschneider’s	MVR	Right anterolateral thoracotomy
	St Thomas’		
Vistarini N^15^	Del Nido	AVR	Mini-sternotomy
	Whole blood		
Vivacqua A^11^	Custodiol	AVR	Does not specify
	Whole blood		
Zizadeh D^16^	Del Nido	AVR	Mini-sternotomy and Right anterolateral thoracotomy
	Whole blood		

The table shows the cardioplegia(s) used in each study, the type of minimally invasive surgery and the surgical approach.

MVR: mitral valve repair; AVR: aortic valve replacement.

## Summary of results

To understand the discussion regarding the safety of different cardioplegic solutions for MIVS, it is worth noting the key differences between the main solutions. Custodiol/Bretschneider’s cardioplegia, also called HTK solution, is a crystalloid cardioplegia that includes a combination of histidine (pH buffer), tryptophan (membrane potential stabiliser) and alpha ketoglutarate (precursor for nicotinamide adenine dinucleotide (NAD), important in ATP synthesis).^17^ DNC utilizes a combination of lidocaine (sodium channel blockade), mannitol (regulates osmotic pressure) and a crystalloid to blood ratio of 4:1. It was first developed for use in paediatric cardiac surgery but is now widely used in adult procedures. On top of this, a single dose at 20 ml/kg can provide myocardial arrest for up to 90 minutes.^18^ Whole blood cardioplegia has higher oxygen carrying capacity than crystalloid, reducing risk of ischaemic damage by allowing the myocardium to aerobically produce ATP and NAD. It is also thought to be safer by providing a “biological” pH buffer.^1^

St Thomas’ Cardioplegia solution is most commonly utilized as BC that has been diluted 4:1 (blood:saline), but is a solution that can also be used in crystalloid solutions. With the aim of making the solution as physiological as possible, cause cardiac arrest and trying to avoid cardiac damage due to a low pH (a problem associated with original cardioplegic solutions), but with a Potassium concentration that is higher than normal serum concentrations to cause cardiac arrest along with the addition Sodium channel blockers (e.g. Lidocaine, Procaine).^19^ The use of sodium channel blockers was originally for its membrane stabilizing effects, but has since been shown to significantly reduce ventricular fibrillation and markers of cardiac damage (in the form of enzymes) post-surgery.^20^ Unlike other cardioplegia solutions, the inclusion of Magnesium chloride in St Thomas’ cardioplegia has been shown to have a beneficial effects against calcium overload during myocardial ischaemia following reperfusion.^21^ The solution has undergone different configurations, with St Thomas’ 2 being developed in 1981.^19^

Whole blood cardioplegia (WBC) can be delivered either cold or warm, with cold blood cardioplegia reducing metabolic activity of the myocardium by slowing enzyme activity; however its use is thought to delay recovery post-operatively.^22^ Whole blood cardioplegia can provide a physiological buffer to the acidosis that occurs to the myocardium during arrest and provides oxygen for the arrested myocardium. The inclusion of additives to the solution (e.g. ion channel blockers and local anaesthetics) also improve the ability of the cardioplegia to cause arrest, provide greater cardioprotection and lead to better recovery after surgery.^23^

**Figure 2.**
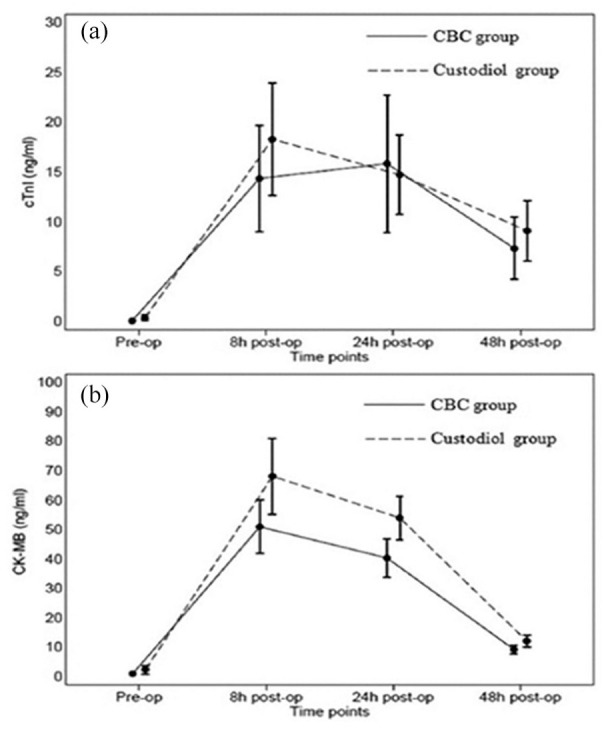
Comparing Bretschneider’s cardioplegia to St Thomas’s cardioplegia for Troponin-I (a) and CK-MB (b). Through all the measurements, there was no significant difference between St Thomas’ cardioplegia or Bretschneider’s for either Troponin-I or CK-MB.

Ideally, to determine the best cardioplegia, comparisons within the same institute should be made. The most recent research investigating the safety of Custodiol in MIVS was done by Vivacqua et al, who randomly assigned patients undergoing minimally invasive aortic valve replacement (MIAVR) to either Custodiol or WBC groups.^11^ Whilst the data results have not been quoted, TnT-I and CK-MB of both groups were published as a ratio of the means. It was found that the TnI mean for Custodiol was 76.8% of the WBC group and the ratio of the means of CK-MB was 0.847, with Custodiol being lower. Whilst Custodiol produced lower TnI and CK-MB post-operatively across all times, these results were not significantly different. Although this work doesn’t show the measurements for TnI and CK-MB, there is evidence for Custodiol’s safety. Mork et al. compared Bretschneider to St Thomas’ 2 solution for minimally invasive mitral valve surgery (MIMVS) revealed a mixed picture.^7^ Certainly, their primary outcome (peak TnT post-operatively) showed St Thomas’ solution was safer than Bretschneider (561 and 716 ng/L respectively), but secondary outcomes and other markers used (such as CK-MB) did not produce different measurements (max-CK-MB Bretschneider = 40.0 ug/L, St Thomas’ solution = 33.4 ug/L). There are mitigating factors to consider; despite case-matching there were differences in group size for both groups and the TnT and CK-MB measurements were not done at specified intervals post-operatively. Despite this, this study appears to suggest comparable safety for St Thomas’ and Bretschneider cardioplegia. Work by De Palo et al set out to compare the use of Bretschneider’s to St Thomas’ cardioplegia for a variety of MIVS.^12^ TnI and CK-MB were measured pre-operatively and 8, 24 and 48 hours post-operatively. For TnI and CK-MB measurements, there was found to be no significant difference across any of the time periods measured (Figure 2) and the mean values were not significantly different (Bretschneider; mean TnI = 21 ± 47 ng/mL, mean CK-MB = 73 ± 84 ng/mL. St Thomas’ cardioplegia; mean TnI = 18 ± 46 ng/mL, mean CK-MB = 53 ± 61 ng/mL). Further analysis revealed that aortic cross clamp time was the most important factor for TnI and CK-MB release. Following the transition from full sternotomy and WBC to MICS (via right mini-thoracotomy) and Custodiol, Matzelle et al performed a retrospective study of their results.^13^ Whilst TnI was not used as a primary outcome (length of stay in ITU and hospital stay duration), their results for peak TnI within the first 24 hours post-op from the first 100 patients undergoing MIMVS was 5.1 ug/L (range from 0.8 to 40 ug/L).

Whilst there is evidence for safety for crystalloid cardioplegia, it is worth investigating how mixed blood cardioplegias compare. A review of the work of a single operating team compared the use of BC St Thomas’ and DNC solutions when doing MIAVR, MIMVS and dual valve MIVS. This review reported no significant difference in these biomarkers post-operatively, as post-operative TnT levels for DNC and St Thomas’ solution were 0.39 and 0.36 ng/mL and BNP levels were 309 and 285 pg/mL respectively.^14^ Vistarini et al compared the use of DNC and “standard” blood cardioplegia (SBC) in MIAVR via mini-sternotomy.^15^ At day 1 and 2 post-operatively, TnT and CK-MB levels for DNC and SBC were not different (DNC TnT; 205 ng/L ± 98 and 157 ng/L ± 79, CK-MB; 12.1 ug/L ± 4.6, 7.4 ug/L ± 6.0. SBC TnT; 255 ng/L ± 76 and 180 ng/L ± 64, CK-MB; 16.4 ug/L ± 6.4, 12.7 ug/L ± 4.7), however there was a significant difference in CK-MB levels immediately after the operation (DNC = 11.4 ug/L ± 5.4, SBC = 17.7 ug/L ± 6.9, p = 0.004). However, it is worth noting that these values were not significantly different at day 1 post-operatively.

The study by Ziazadeh et al did not reveal any difference in Tn-T levels post-MIAVR between DNC and SBC groups (0.44 ng/mL ± 1.7 and 0.3 ng/mL ± 0.29 respectively).^16^ However, it did reveal there was a significant reduction in the number of doses and volume of cardioplegia used, with the DNC group receiving fewer doses. The author discusses the fact that, with reduced dosing, the cardioplegia regimen and therefore the operation is less complicated. These same findings and conclusions have been discussed in other studies comparing DNC to cold-WBC, with DNC use “improving surgical efficiency” without any significant difference in Tn-T levels post-operatively for 4 days.^15,24^ Vivacqua et al also discusses the benefit of the increased duration of arrest for Custodiol.^11^ Whilst they found no benefit compared to blood cardioplegia (in terms of biomarkers), they discuss that repeated dosing with blood cardioplegia “interrupts the technical flow of surgery”. The benefits of prolonged arrest by Bretschneider’s are also discussed, again pointing out that repeating dosage in MIVS leads to disruption in the operation and may require the insertion of a retrograde catheter (which would prolong surgical time).^13^

## Discussion

The aim of this paper was to establish whether a specific cardioplegia solution provides better myocardial protection during MIVS. Throughout the literature, there appears to be no significant difference in post-operative myocardial damage between the different solutions used. The lack of difference in myocardial damage post-MIVS was shown when comparing DNC to SBC, DNC to St Thomas’ cardioplegia and Custodiol to WBC or St Thomas’ solution in MIVS.^7,11–16,24^ When analysing the results, only Mork et al. found a significant difference in TnI, however the group size for Bretschneider’s was significantly larger than that of St Thomas’s solution in that study.^7^ As cardiac arrest is required to perform the procedures in a safe manner, there is always a risk of ischaemic damage. The notion that the best cardioplegic solution for myocardial protection is yet to be found has been supported in several reviews.^25,26^ It can be concluded that current cardioplegic solutions are equally effective at protecting the myocardium during MIVS. For all practical purposes, the use of BC or CC comes down to operator preference. Solutions, such as Bretschneider’s and DNC (which provide a longer arrest time than blood cardioplegia and therefore reduce the need for repeated doses) were preferred by two of the authors as they disrupt the flow of the surgery less. This was revealed in discussions when the author cited the “simplicity” of single-dose cardioplegia, compared to cardioplegia that required multiple doses, is why DNC and Bretschneider’s solutions are used.^27,28^ The volume of crystalloid solution added during myocardial protection varies tremendously as a function of the cardioplegia type. The use of ultrafiltration ameliorates the effects of hemodilation and occurs more than twice as often as high crystalloid solutions, such as DN and HTK, are used. The administration of HTK solution in patients receiving MIVS results in marked decline in serum sodium. However, serum osmolality remained stable during surgery indicating presence of isotonic hyponatraemia not requiring treatment. In fact, correction of hyponatraemia during/after cardioplegia with HTK solution might cause hypertonicity with its associated adverse events.

There may be an argument that, until the next breakthrough in cardioplegia is made, the level of myocardial protection that can be provided has currently reached a plateau.^29^ A study that reviewed right ventricular (RV) function following MIMVS with three groups; groups 1 and 2 underwent sternotomy (with Blood and Custodiol cardioplegia respectively) and group 3 underwent a minimally invasive approach using Custodiol. Interestingly, there was no differences observed between Groups 1 and 2 post-operatively, but there was improved RV function for group 3 compared with group 2, pointing towards the idea that the surgical route used and not the cardioplegia result in the degree of cardiac damage.^30^ In this study, an improved myocardial function was found with the minimally invasive group, despite no significant differences in the total cross-clamp or cardiopulmonary bypass times. There is no evidence in the literature so far that the need for intra-aortic balloon pump and/or circulatory support devices and the requirement for permanent pacemakers is higher with BC or CC. The advances in robotic surgery may provide the next big break through. Whilst there is limited information regarding its safety due to cost and lack of training with the robotic tools, as some surgeries can be performed on a still-beating heart (via organ mounting), this may make cardiac arrest redundant and provide better protection.^31^

A limitation of this work is the heterogeneity in the criteria used, as many research papers use post-operative myocardial infarction/mortality rates as their primary outcomes and lack post-operative biomarkers. Whilst the sources included have great variety in the TnI/TnT/CK-MB levels recorded, this can still be used as a reliable method for comparing myocardial protection post-operatively, whereas using mortality rates doesn’t reveal whether patients died as a complication of surgery or inadequate myocardial protection. The limited use of biomarkers to reveal myocardial damage has been observed in other reviews on the topic.^26^ The lack of sources that emerged from the literature search shows that more work must be done to establish whether a specific cardioplegic solution provides better myocardial protection compared to others for MIVS.

Current evidence regarding the safety of cardioplegic solutions for MICS has shown that there is no evidence concluding which is the safest solutions. Preference for cardioplegia use in MICS is determined by surgeon preference, but solutions that cause longer cardiac arrest per infusion have been noted to be preferred due to making procedures simpler.
